# Maternal polyunsaturated fatty acids and allergic disease development in the offspring

**DOI:** 10.1111/pai.13876

**Published:** 2022-11-11

**Authors:** Ruyu Li, Hui Xing Lau, Qai Ven Yap, Yiong Huak Chan, Elizabeth Huiwen Tham, Anne Eng Neo Goh, Hugo Van Bever, Johan Gunnar Eriksson, Shiao‐Yng Chan, Kok Hian Tan, Yap Seng Chong, Bee Wah Lee, Lynette Pei‐chi Shek, Fabian Kok Peng Yap, Philip C. Calder, Keith M. Godfrey, Mary Foong‐Fong Chong, Evelyn Xiu Ling Loo

**Affiliations:** ^1^ Singapore Institute for Clinical Sciences Agency for Science, Technology and Research Singapore Singapore; ^2^ Department of Biostatistics, Yong Loo Lin School of Medicine National University of Singapore Singapore Singapore; ^3^ Department of Paediatrics, Yong Loo Lin School of Medicine National University of Singapore Singapore Singapore; ^4^ Khoo Teck Puat‐National University Children's Medical Institute National University Hospital, National University Health System Singapore Singapore; ^5^ Human Potential Translational Research Programme, Yong Loo Lin School of Medicine National University of Singapore Singapore Singapore; ^6^ Allergy Service, Department of Paediatrics KK Women's and Children's Hospital Singapore Singapore; ^7^ Department of Obstetrics & Gynaecology, Yong Loo Lin School of Medicine National University of Singapore and National University Health System Singapore Singapore; ^8^ Folkhälsan Research Center Helsinki Finland; ^9^ Department of General Practice and Primary Health Care University of Helsinki Helsinki Finland; ^10^ Department of Maternal Fetal Medicine KK Women's and Children's Hospital (KKH) Singapore Singapore; ^11^ Duke‐NUS Medical School Singapore Singapore; ^12^ Lee Kong Chian School of Medicine Nanyang Technological University Singapore Singapore; ^13^ Endocrinology Service, Department of Paediatrics KK Women's and Children's Hospital Singapore Singapore; ^14^ School of Human Development and Health, Faculty of Medicine University of Southampton Southampton UK; ^15^ NIHR Southampton Biomedical Research Centre University Hospital Southampton NHS Foundation Trust and University of Southampton Southampton UK; ^16^ MRC Lifecourse Epidemiology Unit, Faculty of Medicine University of Southampton Southampton UK; ^17^ Saw Swee Hock School of Public Health National University of Singapore Singapore Singapore

**Keywords:** allergic sensitization, eczema, polyunsaturated fatty acids, rhinitis, wheeze

To the Editor,

The increasing global prevalence of allergic diseases makes it imperative to identify modifiable risk factors for allergic disease development. Maternal antenatal plasma fatty acid composition has been proposed as a risk factor of infant allergic disease.^1^, ^2^ Polyunsaturated fatty acids (PUFAs) are key components of cell membranes and influence immune cell function by regulating membrane fluidity, intracellular signaling, and gene expression.^3^ They can be classified into n‐3 and n‐6 PUFAs, which are linked to production of anti‐inflammatory and pro‐inflammatory molecules, respectively.^3^ The fetoplacental unit lacks the desaturase enzymes required to synthesize long chain PUFAs so that, during pregnancy, the fetus is dependent on transplacental supply of PUFAs from the mother.^4^

Only three studies conducted in European countries have examined the association of the ratio of PUFA precursors to products in the maternal bloodstream (e.g., in plasma phospholipids) with offspring allergic disease development and these have reported conflicting results.^1^, ^2^, ^5^ Dietary n‐3 PUFA alpha‐linolenic acid (ALA) undergoes desaturation to form longer chain n‐3 PUFAs, mainly eicosapentaenoic acid (EPA) and docosahexaenoic acid (DHA).^3^ Similarly, dietary n‐6 PUFA linolenic acid (LA) competes for the same enzymes to form arachidonic acid (AA).^3^ These metabolites are further converted into immunomodulatory oxylipin mediators such as eicosanoids.^3^ Through their effects on the cell membrane, cell signaling, gene expression, and oxylipin production, PUFAs can influence production of cytokines involved in allergic disease. The ratios of ALA and LA to their respective unsaturated products indicate metabolism efficiency of the precursors.^1^ The balance of n‐3 to n‐6 PUFAs as well as PUFA precursors to their products may influence the risk of allergic disease development.

In this study, we investigated long‐term associations between maternal PUFAs in plasma phospholipids during pregnancy and the risk of offspring rhinitis, wheeze, eczema, and allergic sensitization up to age 8 years in the Growing Up in Singapore Towards healthy Outcomes (GUSTO) cohort. We hypothesized that higher n‐3 metabolites is protective against inflammation and allergy and that higher total n‐3:total n‐6 PUFAs, higher (DHA + EPA):AA and LA:AA ratios and lower ALA:(EPA + DHA) during pregnancy are associated with decreased pro‐inflammatory cord blood cytokines at birth and consequently decreased risk of offspring allergic diseases in the first 8 years of life.

Demographic data were gathered by interviewer‐administered questionnaires. Offspring allergic outcomes were evaluated via modified International Study of Asthma and Allergies in Childhood (ISAAC) questionnaires (Appendix S1). Offspring allergic sensitization was determined by skin prick testing at ages 18, 36 months, 5 and 8 years for common allergens in Singapore (Appendix S2). Maternal plasma phospholipids at gestational week 26 were measured using gas chromatography–mass spectrometry (Appendix S3) and infant cord blood cytokines were assayed using modified Luminex assay via DropArray multiplex assay (Appendix S4). Ethics approval was obtained from the Domain Specific Review Board of Singapore National Healthcare Group (D/2009/021) and the Centralized Institutional Review Board of SingHealth (2018/2767). Analyses were performed using the Statistical Package for the Social Sciences, Version 27 (IBM Cooperation, New York) (Appendix S5).

After removing subjects with missing data on maternal plasma PUFAs concentrations and offspring allergic outcomes, 920 mother–offspring pairs were included in the study (Table 1). There were no differences between included and excluded participants except there was a higher proportion of nulliparous women among the excluded participants (Table S1).

**TABLE 1:** Demographics of study population

	*n*	Median (IQR) or *n* (%)
Ethnicity	920	
Chinese		501 (54.5%)
Indian		171 (18.6%)
Malay		247 (26.8%)
Maternal allergy history	920	
Yes		352 (38.3%)
No		543 (60.7%)
Mother's educational attainment	920	
Post‐secondary and higher		635 (69.9%)
Secondary school education or less		273 (30.1%)
Maternal exposure to smoke during pregnancy week 26	920	
Yes		337 (38.3%)
No		543 (61.7%)
Parity	920	
Parous		533 (57.9%)
Nulliparous		387 (42.1%)
Mode of delivery	920	
Vaginal delivery		647 (70.3%)
Caesarean section		273 (29.7%)
Mother's age at delivery (years)	920	31.0 (27.5–34.8)
Sex of offspring	920	
Female		439 (47.7%)
Male		481 (52.3%)
Feeding practices	877	
Mainly formula		383 (43.7%)
Mainly breastfeeding		110 (12.5%)
Mixed		384 (43.8%)
Maternal plasma PUFA ratios during pregnancy		
ALA:(DHA + EPA)	920	0.03 (0.02–0.05)
LA:AA	920	2.80 (2.29–3.37)
ALA:LA	920	0.01 (0–0.01)
(DHA + EPA):AA	920	0.67 (0.54–0.84)
Total n‐3 PUFAs (mcg/ml)	920	140.15 (101.82–199.08)
Total n‐6 PUFAs (mcg/ml)	920	794.04 (621.39–1006.95)
Total n‐3:total n‐6 PUFAs ratio	920	0.18 (0.14–0.22)
Total PUFAs (mcg/ml)	920	941.96 (733.07–1192.02)
Cord blood cytokines at birth (pg/ml)		
IL‐10	646	0.88 (0.61–1.32)
IL‐6	634	2.91 (1.70–6.23)
TNF‐α	647	3.64 (3.14–4.21)
Eotaxin	693	57.36 (39.29–89.43)
IL‐1RA	670	373.81 (239.53–668.35)
IP‐10	693	68.04 (49.13–97.91)
MCP‐1	690	99.76 (69.90–146.96)
MIG	642	12.66 (8.37–18.55)
MIP‐1alpha	689	6.40 (4.75–8.46)
MIP‐1beta	643	26.14 (18.43–38.54)
VEGF‐A	693	598.53 (433.53–1050.20)
IL‐12p40	693	301.76 (219.69–378.34)
PAI‐1	693	5874.48 (4842.90–7124.85)
CRP	691	17,425.79 (11,490.11–25,195.13)
Rhinitis by 18 months	750	
Yes		396 (52.8%)
No		354 (47.2%)
Rhinitis by 36 months	748	
Yes		472 (63.1%)
No		276 (36.9%)
Rhinitis by 5 years	724	
Yes		491 (67.8%)
No		233 (32.2%)
Rhinitis by 8 years	798	
Yes		515 (64.5%)
No		283 (35.5%)
Wheeze by 18 months	672	
Yes		97 (14.4%)
No		575 (85.6%)
Wheeze by 36 months	651	
Yes		169 (26.0%)
No		482 (74.0%)
Wheeze by 5 years	601	
Yes		211 (35.1%)
No		390 (64.9%)
Wheeze by 8 years	703	
Yes		226 (32.1%)
No		477 (67.9%)
Eczema by 18 months	705	
Yes		156 (22.1%)
No		549 (77.9%)
Eczema by 36 months	666	
Yes		193 (29.0%)
No		473 (71.0%)
Eczema by 5 years	611	
Yes		207 (33.9%)
No		404 (66.1%)
Eczema by 8 years	715	
Yes		233 (32.6%)
No		482 (67.4%)
Sensitization by 18 months	759	
Yes		106 (14.0%)
No		653 (86.0%)
Sensitization by 36 months	700	
Yes		211 (30.1%)
No		489 (69.9%)
Sensitization by 5 years	661	
Yes		324 (49.0%)
No		337 (51.0%)
Sensitization by 8 years	668	
Yes		472 (70.7%)
No		196 (29.3%)

In multivariate Poisson analysis with adjustment for maternal age, history of allergy, parity, smoke exposure during pregnancy, educational attainment, mode of delivery, offspring's sex, breastfeeding practices, and offspring fish oil intake (DHA + EPA):AA (adjRR = 2.2, 95% CI = 1.1–4.3) and total n‐3:total n‐6 PUFAs (adjRR = 2.3, 95% CI = 1.1–4.9) increased the risk of wheeze by 18 months (Table 2). In stratified analyses by exposure to allergic sensitization by 18 months, these associations were only demonstrated in non‐atopic children [(DHA + EPA):AA (adjRR = 2.9, 95% CI = 1.3–6.7) and n‐3:n‐6 PUFA ratios (adjRR = 3.3, 95% CI = 1.3–8.2)]. No associations were observed between maternal ALA:(DHA + EPA), LA:AA, ALA:LA, total n‐3 PUFAs, total n‐6 PUFAs and total PUFAs and other allergic outcomes by 8 years.

**TABLE 2:** Multivariate analysis on maternal blood plasma PUFA to metabolite ratios and total PUFA concentrations during pregnancy and offspring allergic rhinitis, wheeze with use of nebulizers, eczema, and sensitization by 18, 36 months, 5 and 8 years of age

	Month 18			Month 36			Year 5			Year 8		
	*n*	RR (95% CI)	*p*‐value^a^	*n*	RR (95% CI)	*p*‐value^b^	*n*	RR (95% CI)	*p*‐value^c^	*n*	RR (95% CI)	*p*‐value^c^
Allergic rhinitis												
Ln(ALA:(DHA + EPA))	686	0.92 (0.79–1.08)	.318	643	0.94 (0.81–1.09)	.408	537	0.92 (0.78–1.08)	.300	588	0.94 (0.81–1.1)	.424
Ln(LA:AA)	686	1.1 (0.8–1.6)	.550	643	1.1 (0.8–1.5)	.627	537	1.1 (0.7–1.6)	.653	588	1.2 (0.8–1.7)	.440
Ln(ALA:LA)	686	0.94 (0.81–1.10)	.463	643	0.95 (0.82–1.11)	.534	537	0.92 (0.78–1.08)	.319	588	0.93 (0.8–1.09)	.381
Ln((DHA + EPA):AA)	686	1.2 (0.9–1.6)	.284	643	1.1 (0.8–1.5)	.399	537	1.1 (0.8–1.5)	.611	588	1.1 (0.8–1.5)	.575
Ln(Total n‐3:total n‐6 PUFAs)	686	1.1 (0.8–1.5)	.618	643	1.1 (0.8–1.5)	.697	537	1.0 (0.7–1.4)	.943	588	1.0 (0.7–1.4)	.985
Ln(Total n‐3 PUFAs)	686	1.0 (0.8–1.3)	.922	643	1.0 (0.8–1.2)	.960	537	0.96 (0.77–1.21)	.757	588	1.0 (0.8–1.3)	.943
Ln(Total n‐6 PUFAs)	686	0.95 (0.69–1.30)	.745	643	0.96 (0.71–1.29)	.766	537	0.92 (0.67–1.27)	.618	588	1.0 (0.7–1.4)	.907
Ln(Total PUFAs)	686	0.96 (0.71–1.30)	.788	643	0.96 (0.72–1.29)	.806	537	0.92 (0.67–1.27)	.627	588	1.0 (0.7–1.4)	.916
Wheeze with nebulizers												
Ln(ALA:(DHA + EPA))	620	0.87 (0.62–1.22)	.433	586	0.93 (0.71–1.22)	.616	479	0.97 (0.74–1.26)	.795	550	0.96 (0.76–1.22)	.742
Ln(LA:AA)	620	1.1 (0.5–2.4)	.754	586	1.1 (0.6–2.0)	.767	479	1.0 (0.5–1.8)	.988	550	1.2 (0.7–2.1)	.557
Ln(ALA:LA)	620	1.0 (0.7–1.5)	.779	586	0.97 (0.74–1.26)	.793	479	1.0 (0.8–1.3)	.917	550	0.98 (0.76–1.25)	.840
Ln((DHA + EPA):AA)	620	2.2 (1.1–4.3)	**.019**	586	1.2 (0.7–2.0)	.482	479	1.2 (0.7–2.0)	.494	550	1.2 (0.7–2.0)	.434
Ln(Total n‐3:total n‐6 PUFAs)	620	2.3 (1.1–4.9)	**.023**	586	1.1 (0.6–2.0)	.661	479	1.2 (0.7–2.1)	.469	550	1.1 (0.7–1.9)	.693
Ln(Total n‐3 PUFAs)	620	1.4 (0.9–2.3)	.151	586	0.92 (0.64–1.33)	.671	479	0.97 (0.68–1.39)	.883	550	0.92 (0.65–1.29)	.622
Ln(Total n‐6 PUFAs)	620	0.97 (0.50–1.87)	.930	586	0.77 (0.46–1.28)	.319	479	0.81 (0.49–1.32)	.391	550	0.77 (0.48–1.25)	.294
Ln(Total PUFAs)	620	1.1 (0.6–2.1)	.791	586	0.79 (0.48–1.31)	.362	479	0.83 (0.51–1.35)	.464	550	0.79 (0.50–1.27)	.332
Eczema												
Ln(ALA:(DHA + EPA))	645	1.1 (0.8–1.3)	.687	601	0.97 (0.77–1.22)	.805	491	1.0 (0.8–1.3)	.856	559	1.0 (0.8–1.3)	.693
Ln(LA:AA)	645	1.6 (0.9–2.8)	.119	601	1.3 (0.8–2.3)	.308	491	1.3 (0.7–2.4)	.362	559	1.3 (0.7–2.2)	.376
Ln(ALA:LA)	645	1.0 (0.8–1.3)	.833	601	0.98 (0.78–1.24)	.886	491	1.0 (0.8–1.3)	.842	559	1.1 (0.9–1.3)	.568
Ln((DHA + EPA):AA)	645	1.3 (0.8–2.1)	.348	601	1.3 (0.8–2.0)	.298	491	1.2 (0.7–2.0)	.430	559	1.3 (0.8–2.1)	.274
Ln(Total n‐3:total n‐6 PUFAs)	645	1.1 (0.6–1.8)	.842	601	1.1 (0.7–1.8)	.616	491	1.1 (0.6–1.8)	.773	559	1.2 (0.7–1.9)	.546
Ln(Total n‐3 PUFAs)	645	1.0 (0.7–1.4)	.894	601	1.1 (0.8–1.5)	.601	491	1.1 (0.8–1.6)	.572	559	1.3 (0.9–1.8)	.148
Ln(Total n‐6 PUFAs)	645	1.0 (0.6–1.6)	.998	601	1.1 (0.7–1.7)	.790	491	1.1 (0.7–1.8)	.600	559	1.4 (0.9–2.2)	.142
Ln(Total PUFAs)	645	1.0 (0.6–1.6)	.969	601	1.1 (0.7–1.7)	.747	491	1.1 (0.7–1.8)	.588	559	1.4 (0.9–2.2)	.131
Sensitization												
Ln(ALA:(DHA + EPA))	681	1.0 (0.8–1.3)	.953	624	0.97 (0.78–1.21)	.812	533	1.0 (0.8–1.2)	.933	523	0.97 (0.84–1.13)	.710
Ln(LA:AA)	681	1.1 (0.5–2.0)	.873	624	0.87 (0.53–1.43)	.578	533	1.0 (0.7–1.6)	.846	523	0.95 (0.66–1.36)	.777
Ln(ALA:LA)	681	1.1 (0.8–1.4)	.714	624	1 (0.8–1.3)	.923	533	1.0 (0.9–1.2)	.786	523	0.98 (0.84–1.15)	.828
Ln((DHA + EPA):AA)	681	1.2 (0.7–2.2)	.459	624	1 (0.7–1.6)	.854	533	1.1 (0.8–1.6)	.579	523	1.0 (0.7–1.4)	.934
Ln(Total n‐3:total n‐6 PUFAs)	681	1.3 (0.7–2.3)	.480	624	1.2 (0.7–1.8)	.554	533	1.1 (0.7–1.7)	.616	523	1.0 (0.7–1.4)	.831
Ln(Total n‐3 PUFAs)	681	1.0 (0.7–1.6)	.888	624	1.1 (0.8–1.5)	.486	533	1.1 (0.9–1.5)	.414	523	1.0 (0.8–1.2)	.988
Ln(Total n‐6 PUFAs)	681	0.86 (0.47–1.58)	.623	624	1.1 (0.7–1.7)	.663	533	1.1 (0.8–1.7)	.492	523	0.96 (0.70–1.32)	.823
Ln(Total PUFAs)	681	0.89 (0.49–1.61)	.697	624	1.1 (0.7–1.7)	.620	533	1.1 (0.8–1.7)	.459	523	0.97 (0.71–1.32)	.856

*Note*: Benjamini‐Hochberg correction with false discovery rate at 0.40 and *n* = 32 was applied to each outcomes and significant *p*‐value in bold.

^a^ Adjusted for maternal age at delivery, history of allergy, parity, educational attainment, smoke exposure during pregnancy, mode of delivery, breastfeeding practices, and offspring's sex.

^b^ Adjusted for maternal age at delivery, history of allergy, parity, educational attainment, smoke exposure during pregnancy, mode of delivery, breastfeeding practices, offspring's sex, and year 3 fish oil intake.

^c^ Adjusted for maternal age at delivery, history of allergy, parity, educational attainment, smoke exposure during pregnancy, mode of delivery, breastfeeding practices, offspring's sex, and year 5 fish oil intake.

We next determined if maternal plasma (DHA + EPA):AA and total n‐3:total n‐6 PUFAs were related to cord blood cytokines; only higher total n‐3:total n‐6 PUFAs ratio was negatively associated with eotaxin (adjβ = −0.25, 95% CI = −0.42 to −0.08) and weakly associated with interleukin‐12 subunit p40 (IL‐12p40) (adjβ = −0.12, 95% CI = −0.24 to 0, Table 3). There was no mediation effect by any cord blood cytokine in the associations between (DHA + EPA):AA or total n‐3:total n‐6 PUFAs ratios and offspring allergic disease in mediation analysis (Table S2).

**TABLE 3:** Association between maternal blood plasma PUFA (DHA + EPA):AA and total n‐3:total n‐6 PUFAs ratios during pregnancy and cord blood cytokines

Cytokines (pg/ml)	*n*	*B* (95% CI)	*p*‐value	*n*	*B* (95% CI)^a^	*p*‐value^a^
Ln((DHA + EPA):AA)						
ln(IL‐10)	645	0 (−0.15 to 0.14)	.965	566	−0.03 (−0.19 to 0.13)	.680
ln(IL‐6)	634	0 (−0.23 to 0.22)	.966	556	0 (−0.24 to 0.24)	.983
ln(TNF‐α)	647	−0.04 (−0.09 to 0.01)	.128	568	−0.03 (−0.08 to 0.03)	.341
ln(Eotaxin)	693	−0.17 (−0.31 to −0.02)	.024	614	−0.17 (−0.33 to −0.01)	.033
ln(IL‐1RA)	670	−0.03 (−0.24 to 0.17)	.753	592	−0.09 (−0.32 to 0.14)	.456
ln(IP‐10)	693	0.05 (−0.08 to 0.18)	.458	614	0.06 (−0.09 to 0.20)	.441
ln(MCP‐1)	690	−0.06 (−0.21 to 0.09)	.432	611	−0.09 (−0.25 to 0.08)	.288
ln(MIG)	642	−0.03 (−0.20 to 0.15)	.772	569	−0.01 (−0.2 to 0.18)	.911
ln(MIP‐1alpha)	689	−0.02 (−0.13 to 0.09)	.687	610	−0.03 (−0.15 to 0.09)	.590
ln(MIP‐1beta)	643	−0.01 (−0.16 to 0.14)	.877	568	−0.02 (−0.18 to 0.15)	.834
ln(VEGF‐A)	693	−0.12 (−0.29 to 0.06)	.203	614	−0.13 (−0.33 to 0.06)	.185
ln(IL‐12p40)	693	−0.04 (−0.14 to 0.06)	.459	614	−0.09 (−0.20 to 0.02)	.112
ln(PAI‐1)	693	−0.06 (−0.17 to 0.05)	.293	614	−0.08 (−0.21 to 0.04)	.207
ln(CRP)	691	−0.01 (−0.19 to 0.17)	.887	612	−0.08 (−0.28 to 0.11)	.407
Ln(total n‐3:total n‐6 PUFAs)						
ln(IL‐10)	645	−0.06 (−0.21 to 0.10)	.466	566	−0.10 (−0.28 to 0.07)	.255
ln(IL‐6)	634	−0.18 (−0.43 to 0.06)	.135	556	−0.12 (−0.38 to 0.14)	.381
ln(TNF‐α)	647	−0.04 (−0.10 to 0.01)	.134	568	−0.04 (−0.11 to 0.02)	.159
ln(Eotaxin)	693	−0.2 (−0.36 to −0.05)	.010	614	−0.25 (−0.42 to −0.08)	**.004**
ln(IL‐1RA)	670	‐0.08 (−0.30 to 0.14)	.459	592	−0.15 (−0.40 to 0.10)	.229
ln(IP‐10)	693	0.03 (−0.10 to 0.17)	.640	614	0.02 (−0.14 to 0.17)	.823
ln(MCP‐1)	690	−0.07 (−0.23 to 0.09)	.381	611	−0.12 (−0.30 to 0.06)	.177
ln(MIG)	642	−0.07 (−0.26 to 0.11)	.436	569	−0.08 (−0.29 to 0.13)	.449
ln(MIP‐1alpha)	689	−0.01 (−0.13 to 0.10)	.816	610	−0.06 (−0.19 to 0.07)	.397
ln(MIP‐1beta)	643	−0.06 (−0.22 to 0.11)	.500	568	−0.12 (−0.3 to 0.06)	.182
ln(VEGF‐A)	693	−0.07 (−0.26 to 0.12)	.467	614	−0.10 (−0.32 to 0.11)	.332
ln(IL‐12p40)	693	−0.05 (−0.16 to 0.06)	.352	614	−0.12 (−0.24 to 0)	**.047**
ln(PAI‐1)	693	−0.09 (−0.21 to 0.03)	.138	614	−0.12 (−0.26 to 0.01)	.073
ln(CRP)	691	−0.06 (−0.26 to 0.13)	.531	612	−0.10 (−0.31 to 0.12)	.368

*Note*: Benjamini‐Hochberg correction with false discovery rate at 0.40 and *n* = 14 was applied to adjusted models of each outcomes and significant *p*‐value in bold.

a Adjusted for maternal age at delivery, history of allergy, parity, educational attainment, smoke exposure during pregnancy, mode of delivery, breastfeeding practices, and offspring's sex.

In this study, we observed that maternal plasma ALA:(DHA + EPA) and LA:AA were not associated with the development of offspring allergic diseases by the 8‐year follow‐up. The results are supported by the Generation R study^2^ and Avon Longitudinal Study of Parents and Children^5^ studies. Conversely, the Southampton Women's Survey found that ratios of ALA and LA to their products in maternal plasma phosphatidylcholine associated with the risks of wheeze and skin sensitization at 6 years of age, respectively.^1^

It is possible that downstream metabolites of DHA, EPA, and AA may be key to controlling allergy development as their inflammatory activities may differ from one another. The above studies included all unsaturated metabolic products of ALA and LA in the computation of precursor: metabolite ratios while we only included the major metabolites DHA and EPA and AA, respectively. For example, LA is metabolized to form AA, which in turn produces pro‐inflammatory prostaglandins promoting allergic sensitization and to anti‐inflammatory lipoxins promoting the resolution of allergy.^3^ Thus, the overall effect of an individual PUFA or of groups or ratios of PUFAs is difficult to predict. The effect of maternal PUFAs might also be outweighed by other environmental factors which are more relevant to allergy development in our cohort, such as smoking exposure and childcare center attendance during infancy.^6^

We observed that higher maternal total n‐3:total n‐6 PUFAs and (DHA + EPA):AA ratios were associated with a higher risk of early life wheeze by 18 months, especially in non‐atopic children. This finding is supported by the Southampton Women's Survey which reported that AA was inversely associated with non‐atopic persistent/late wheeze.^1^ We postulate that lower maternal n‐6 PUFA levels may increase susceptibility to infections, especially since wheeze in early life is largely caused by viruses or bacteria rather than allergy development.^7^ In particular, AA has the strongest antibacterial and antiviral effect in the lungs, as compared to other PUFAs, possibly by disrupting the microbial cell membrane integrity.^8^, ^9^ Early exposure to n‐6 PUFAs and AA in utero may promote robust immune system development, which protects against infections later in infancy. Further research is needed to elucidate the underlying mechanisms, with our study suggesting the association between n‐3:n‐6 PUFA and (DHA + EPA):AA ratios and offspring wheeze by 18 months is not mediated by cord blood cytokine concentrations.

Strengths of this study include the long‐term follow‐up of participants and the collection of data on allergic diseases, as well as skin prick testing at multiple timepoints up to age 8 years. The comprehensive profiling of participants in this study allowed adjustment for relevant covariates, including offspring fish oil intake which contains high quantities of n‐3 PUFAs. However, maternal blood was only collected at one timepoint, which may not reflect variations in PUFA levels throughout pregnancy.

We found no convincing evidence to suggest that maternal plasma n‐3 PUFA is protective, nor that n‐6 PUFA increases the risk of offspring allergy development. However, our results suggest that higher n‐3 to n‐6 PUFA ratios may be linked to increased risk of early life wheezing illness.

## AUTHOR CONTRIBUTIONS

Ruyu Li: Conceptualization (Supporting), Writing‐original draft (equal), Writing‐review and editing (equal). Hui Xing Lau: Writing‐original draft (equal), Writing‐review and editing (equal). Qai Ven Yap: Formal analysis (equal), Writing‐original draft (equal), Writing‐review and editing (equal). Yiong Huak Chan: Formal analysis (equal), Writing‐review and editing (equal). Mary Foong‐Fong Chong: Methodology (Equal), Project administration (Equal), Writing‐review and editing (equal). Elizabeth Huiwen Tham: Methodology (Equal), Project administration (Equal), Writing‐review and editing (equal). Anne Eng Neo Goh: Methodology (Equal), Project administration (Equal), Writing‐review and editing (equal). Hugo Van Bever: Methodology (Equal), Project administration (Equal), Writing‐review and editing (equal). Johan Gunnar Eriksson: Methodology (Equal), Project administration (Equal), Writing‐review and editing (equal). Shiao‐Yng Chan: Methodology (Equal), Project administration (Equal), Writing‐review and editing (equal). Kok Hian Tan: Methodology (Equal), Project administration (Equal) Writing‐review and editing (equal). Yap Seng Chong: Methodology (Equal), Project administration (Equal), Writing‐review and editing (equal), Funding acquisition (Lead). Bee Wah Lee: Methodology (Equal), Project administration (Equal) Writing‐review and editing (equal). Lynette Pei‐chi Shek: Methodology (Equal), Project administration (Equal) Writing‐review and editing (equal). Fabian Kok Peng Yap: Methodology (Equal), Project administration (Equal), Writing‐review and editing (equal). Philip C Calder: Methodology (Equal), Project administration (Equal), Writing‐review and editing (equal). Keith M Godfrey: Methodology (Equal), Project administration (Equal), Writing‐review and editing (equal). Evelyn Xiu Ling Loo: Conceptualization (lead), Methodology (lead), Formal analysis (lead), Project administration (lead), Supervision (lead), Writing‐original draft (lead), Writing‐review and editing (equal).

## FUNDING INFORMATION

This research is supported by the Singapore National Research Foundation under its Translational and Clinical Research (TCR) Flagship Programme and administered by the Singapore Ministry of Health's National Medical Research Council (NMRC), Singapore—NMRC/TCR/004‐NUS/2008; NMRC/TCR/012‐NUHS/2014. Additional funding is provided by the Singapore Institute for Clinical Sciences, Agency for Science Technology and Research (A*STAR), Singapore. EH Tham is supported by the National Medical Research Council (NMRC) Transition Award grant (MOH‐TA18nov‐003) from NMRC, Singapore. KM Godfrey is supported by the UK Medical Research Council (MC_UU_12011/4), the National Institute for Health Research (NIHR Senior Investigator [NF‐SI‐0515‐10042] and NIHR Southampton Biomedical Research Centre [IS‐BRC‐1215‐20004]), the European Union (Erasmus+ Programme ImpENSA 598488‐EPP‐1‐2018‐1‐DE‐EPPKA2‐CBHE‐JP) and the British Heart Foundation (RG/15/17/3174).

## CONFLICT OF INTEREST

Godfrey KM has received reimbursement for speaking at conferences sponsored by Nestle. Godfrey KM, Chan SY, and Chong YS are part of an academic consortium that has received research funding from Abbot Nutrition, Nestle, and Danone. All other authors declare no conflict of interest.

### PEER REVIEW

The peer review history for this article is available at https://publons.com/publon/10.1111/pai.13876.

## Supporting information


Table S1–S2

Appendix S1–S5
