# Supplementary material for: Maternal polyunsaturated fatty acids and allergic disease development in the offspring
Source: Pediatr Allergy Immunol. 2022 Nov 11;33(11):e13876. doi: 10.1111/pai.13876 (PMC10946560; doi:10.1111/pai.13876)
Supplement: Supplementary file 1 — Table S1–S2 [file PAI-33-0-s001.docx]

**Table S1:** Comparison of demographics of participants included and excluded in the study

|  | Included  (n=920^1^) | Excluded  (n=317^2^) | P-value |
| --- | --- | --- | --- |
| Ethnicity |  |  | 0.378 |
| Chinese | 501 (54.5%) | 190 (59.9%) |  |
| Indian | 171 (18.6%) | 52 (16.4%) |  |
| Malay | 247 (26.8%) | 75 (23.7%) |  |
| Maternal allergy history |  |  | 0.758 |
| Yes | 352 (39.3%) | 125 (40.3%) |  |
| No | 543 (60.7%) | 185 (59.7%) |  |
| Mother’s educational attainment |  |  | 0.110 |
| Post-secondary and higher | 635 (69.9%) | 203 (65.1%) |  |
| Secondary school education or less | 273 (30.1%) | 109 (34.9%) |  |
| Maternal exposure to smoke during pregnancy week 26 |  |  | 0.594 |
| Yes | 337 (38.3%) | 100 (40.2%) |  |
| No | 543 (61.7%) | 149 (59.8%) |  |
| Parity |  |  | **<0.001** |
| Parous | 533 (57.9%) | 103 (40.9%) |  |
| Nulliparous | 387 (42.1%) | 149 (59.1%) |  |
| Mode of delivery |  |  | 0.758 |
| Vaginal delivery | 647 (70.3%) | 174 (69.3%) |  |
| Caesarean section | 273 (29.7%) | 77 (30.7%) |  |
| Mother’s age at delivery (years) | 31.0 (27.5-34.8) | 32.2 (27.6-35.3) | 0.299 |
| Sex of offspring |  |  | 0.448 |
| Female | 439 (47.7%) | 113 (45.0%) |  |
| Male | 481 (52.3%) | 138 (55.0%) |  |
| Feeding practices |  |  | 0.115 |
| Mainly formula | 383 (43.7%) | 78 (51.3%) |  |
| Mainly breastfeeding | 110 (12.5%) | 12 (7.9%) |  |
| Mixed | 384 (43.8%) | 62 (40.8%) |  |

Statistically significant P-values in bold

Values are [median, IQR] for numerical variables & n (%) for categorical variables.

^1^ not all values add up to 920 due to missing data

^2^ not all values add up to 317 due to missing data

**Table S2:** Mediation effect of cord blood cytokines on the association between maternal blood plasma PUFA DHA+EPA:AA and total n-3:n-6 PUFA ratios during pregnancy and offspring allergic rhinitis, wheeze with use of nebulizer and eczema by 18, 36 months, 5 and 8 years of age

|  | Month 18 | | | | Month 36 | | | | Year 5 | | | | Year 8 | | | |  |
| --- | --- | --- | --- | --- | --- | --- | --- | --- | --- | --- | --- | --- | --- | --- | --- | --- | --- |
|  | n | Indirect effect (95% CI) | P-value^1^ | % mediated | n | Indirect effect (95% CI) | P-value^1^ | % mediated | n | Indirect effect (95% CI) | P-value^1^ | % mediated | n | Indirect effect (95% CI) | P-value^1^ | % mediated | |
| Wheeze with nebulizers | | | | | | | | | | | | | | | | |  |
| Ln((DHA+EPA):AA) | | | | | | | | | | | | | | | | |  |
| ln(IL-10) | 352 | 0.001 (-0.028-0.029) | 0.965 | 0.07% | 337 | 0 (-0.001-0.001) | 0.989 | NA |  | 0 (-0.008-0.008) | 0.966 | 0.36% | 318 | 0 (-0.022-0.023) | 0.965 | NA | |
| ln(IL-6) |  | 0 (-0.022-0.021) | 0.966 | NA |  | 0 (-0.019-0.018) | 0.966 | NA |  | 0 (-0.009-0.009) | 0.966 | NA |  | 0 (-0.012-0.011) | 0.966 | 0.49% | |
| ln(TNFa) |  | 0.023 (-0.047-0.094) | 0.518 | 2.6% |  | 0.010 (-0.038-0.058) | 0.674 | 11.33% |  | -0.004 (-0.053-0.045) | 0.872 | NA |  | -0.004 (-0.050-0.041) | 0.848 | 7.77% | |
| ln(Eotaxin) |  | 0.062 (-0.063-0.188) | 0.329 | 6.69% |  | -0.015 (-0.101-0.072) | 0.735 | NA |  | 0.002 (-0.082-0.085) | 0.971 | 3.16% |  | -0.017 (-0.098-0.065) | 0.687 | 24.21% | |
| ln(IL-1RA) |  | -0.006 (-0.046-0.034) | 0.769 | NA |  | -0.003 (-0.024-0.018) | 0.787 | NA |  | -0.004 (-0.032-0.023) | 0.771 | NA |  | -0.001 (-0.013-0.011) | 0.872 | 1.9% | |
| ln(IP-10) |  | -0.01 (-0.052-0.032) | 0.652 | NA |  | -0.001 (-0.026-0.025) | 0.964 | NA |  | 0.001 (-0.025-0.027) | 0.949 | 1.75% |  | -0.002 (-0.029-0.025) | 0.883 | 3.65% | |
| ln(MCP-1) |  | -0.008 (-0.048-0.033) | 0.710 | NA |  | -0.005 (-0.036-0.025) | 0.731 | NA |  | -0.01 (-0.048-0.027) | 0.591 | NA |  | 0.002 (-0.025-0.029) | 0.893 | NA | |
| ln(MIG) |  | -0.014 (-0.106-0.079) | 0.773 | NA |  | -0.007 (-0.055-0.041) | 0.776 | NA | 284 | -0.002 (-0.02-0.016) | 0.821 | NA |  | -0.005 (-0.038-0.029) | 0.784 | 8.14% | |
| ln(MIP-1alpha) |  | 0.008 (-0.037-0.053) | 0.724 | 0.92% |  | 0.007 (-0.030-0.043) | 0.721 | 7.57% |  | 0.004 (-0.022-0.03) | 0.768 | 7.57% |  | 0.01 (-0.041-0.061) | 0.706 | NA | |
| ln(MIP-1beta) |  | -0.002 (-0.033-0.028) | 0.882 | NA |  | 0 (-0.008-0.008) | 0.934 | 0.42% |  | -0.001 (-0.013-0.012) | 0.897 | NA |  | -0.002 (-0.029-0.025) | 0.881 | 3.72% | |
| ln(VEGF-A) |  | -0.014 (-0.067-0.038) | 0.590 | NA |  | 0 (-0.039-0.039) | 0.995 | 0.15% |  | 0 (-0.038-0.038) | 0.980 | 1% |  | -0.009 (-0.048-0.029) | 0.638 | 14.93% | |
| ln(IL-12p40) |  | 0.008 (-0.025-0.041) | 0.625 | 0.94% |  | 0.004 (-0.019-0.027) | 0.710 | 5.16% |  | 0.003 (-0.017-0.023) | 0.769 | 5.97% |  | 0.001 (-0.017-0.019) | 0.919 | NA | |
| ln(PAI-1) |  | -0.001 (-0.045-0.044) | 0.974 | NA |  | 0.012 (-0.023-0.047) | 0.507 | 12.8% |  | 0.013 (-0.022-0.047) | 0.463 | 21.36% |  | 0.014 (-0.022-0.050) | 0.442 | NA | |
| ln(CRP) |  | 0.002 (-0.031-0.036) | 0.888 | 0.28% |  | 0.003 (-0.038-0.044) | 0.887 | 3.55% |  | 0.002 (-0.02-0.023) | 0.890 | 3.14% |  | 0.002 (-0.02-0.023) | 0.890 | NA | |
| Ln(Total n-3: total n-6 PUFA) | | | | | | | | | | | | | | | | |  |
| ln(IL-10) | 352 | 0.011 (-0.032-0.054) | 0.622 | 1.41% | 337 | 0 (-0.024-0.024) | 0.991 | 0.34% | 284 | 0.003 (-0.023-0.03) | 0.821 | NA | 318 | 0.009 (-0.024-0.042) | 0.597 | NA | |
| ln(IL-6) |  | -0.025 (-0.101-0.05) | 0.512 | NA |  | -0.016 (-0.073-0.041) | 0.577 | 28.97% |  | -0.008 (-0.062-0.047) | 0.785 | 17.47% |  | -0.009 (-0.061-0.042) | 0.723 | 3.42% | |
| ln(TNFa) |  | 0.027 (-0.049-0.103) | 0.482 | 3.48% |  | 0.011 (-0.040-0.062) | 0.668 | NA |  | -0.004 (-0.056-0.048) | 0.882 | 10.06% |  | -0.004 (-0.052-0.044) | 0.875 | 1.44% | |
| ln(Eotaxin) |  | 0.073 (-0.076-0.222) | 0.335 | 8.85% |  | -0.018 (-0.124-0.087) | 0.732 | 31.65% |  | 0.002 (-0.100-0.105) | 0.965 | NA |  | -0.02 (-0.120-0.079) | 0.690 | 7.15% | |
| ln(IL-1RA) |  | -0.012 (-0.06-0.036) | 0.624 | NA |  | -0.007 (-0.040-0.026) | 0.673 | 15.04% |  | -0.010 (-0.048-0.028) | 0.598 | 22.18% |  | -0.002 (-0.030-0.025) | 0.864 | 0.9% | |
| ln(IP-10) |  | -0.005 (-0.038-0.027) | 0.739 | NA |  | 0 (-0.017-0.017) | 0.970 | 0.83% |  | 0.001 (-0.017-0.018) | 0.941 | NA |  | -0.001 (-0.019-0.017) | 0.916 | 0.37% | |
| ln(MCP-1) |  | -0.008 (-0.053-0.038) | 0.739 | NA |  | -0.006 (-0.041-0.029) | 0.738 | 13.18% |  | -0.012 (-0.055-0.03) | 0.576 | 25.4% |  | 0.003 (-0.03-0.036) | 0.863 | NA | |
| ln(MIG) |  | -0.04 (-0.147-0.066) | 0.458 | NA |  | -0.020 (-0.078-0.038) | 0.494 | 33.58% |  | -0.006 (-0.041-0.029) | 0.752 | 13.7% |  | -0.013 (-0.059-0.033) | 0.580 | 4.69% | |
| ln(MIP-1alpha) |  | 0.005 (-0.042-0.053) | 0.823 | 0.72% |  | 0.004 (-0.031-0.039) | 0.824 | NA |  | 0.002 (-0.020-0.025) | 0.838 | NA |  | 0.006 (-0.046-0.058) | 0.820 | NA | |
| ln(MIP-1beta) |  | -0.015 (-0.074-0.044) | 0.614 | NA |  | 0.002 (-0.030-0.033) | 0.924 | NA |  | -0.004 (-0.038-0.030) | 0.817 | 10.13% |  | -0.009 (-0.051-0.032) | 0.657 | 3.46% | |
| ln(VEGF-A) |  | -0.006 (-0.039-0.027) | 0.730 | NA |  | 0 (-0.024-0.024) | 0.983 | NA |  | 0 (-0.023-0.024) | 0.983 | NA |  | -0.006 (-0.035-0.022) | 0.651 | 2.41% | |
| ln(IL-12p40) |  | 0.009 (-0.03-0.049) | 0.636 | 1.24% |  | 0.006 (-0.024-0.036) | 0.688 | NA |  | 0.004 (-0.022-0.031) | 0.759 | NA |  | 0.002 (-0.023-0.026) | 0.899 | NA | |
| ln(PAI-1) |  | 0 (-0.066-0.067) | 0.989 | 0.06% |  | 0.017 (-0.030-0.064) | 0.467 | NA |  | 0.019 (-0.026-0.065) | 0.401 | NA |  | 0.021 (-0.025-0.067) | 0.368 | NA | |
| ln(CRP) |  | 0.012 (-0.033-0.057) | 0.599 | 1.58% |  | 0.014 (-0.034-0.062) | 0.571 | NA |  | 0.007 (-0.025-0.040) | 0.656 | NA |  | 0.007 (-0.025-0.040) | 0.661 | NA | |

^1^Adjusted for maternal age of delivery, allergy status, parity, educational attainment, smoke exposure during pregnancy, type of delivery, breastfeeding status and offspring’s sex.

^2^Adjusted for maternal age of delivery, allergy status, parity, educational attainment, smoke exposure during pregnancy, type of delivery, breastfeeding status, offspring’s sex and year 3 fish oil intake.

^3^Adjusted for maternal age of delivery, allergy status, parity, educational attainment, smoke exposure during pregnancy, type of delivery, breastfeeding status, offspring’s sex and year 5 fish oil intake.

Benjamini-Hochberg correction with false discovery rate at 0.40 and n=112 was applied to each outcomes

NA: % mediated is not computed due to inconsistent mediation
