# Supplementary material for: Maternal polyunsaturated fatty acids and allergic disease development in the offspring
Source: Pediatr Allergy Immunol. 2022 Nov 11;33(11):e13876. doi: 10.1111/pai.13876 (PMC10946560; doi:10.1111/pai.13876)
Supplement: Supplementary file 2 — Appendix S1–S5 [file PAI-33-0-s002.docx]

**Appendix S1:** Offspring allergic outcome questionnaires

Offspring allergic outcomes were collected via questionnaires at 3, 6, 12, 15, 18, 24, 36 months, 4, 5, 6, 7 and 8 years of age. Questionnaires were based on the International Study of Asthma and Allergies in Childhood (ISAAC) modified questionnaire. Rhinitis was defined by a positive response to the “Has your child had running nose, blocked or congested nose, snoring or noisy breathing during sleep or when awake that has lasted for two or more weeks duration?”. Wheeze with the use of nebulizers was defined by positive responses to “Has your child ever wheezed?” and “Has your child ever been prescribed with nebulizer/inhaler treatment?”. Eczema was defined by a positive response to the “Has your child ever been diagnosed with eczema?”. Cumulative allergy responses were tabulated where any positive response by that time point defined a positive response, while a negative response at all time points defined a negative response. A maximum of two missing responses was allowed for cumulative allergic outcomes by 8 years if response was negative at 8 years. Informed consent was obtained from all participants.

**Appendix S2:** Skin prick test for allergic sensitization

Allergens tested for were cow's milk, egg, peanut and house dust mites, *Dermatophagoides pteronyssinus*, *Dermatophagoides farina* (Greer Laboratories, Lenoir, NC, USA) and *Blomia tropicalis* (developed in-house) [1] at 18 and 36 months; and all of the above plus crab and shrimp (Greer Laboratories, Lenoir, NC, USA) at 5 and 8 years. At 8 years, SPT for cockroach allergens, *Blattella germanica* and *Periplanata americana,* were also performed*.* SPT was defined as positive at a time point if any of the allergens tested positive at the time point with an average wheal size of at least 3mm.

**Appendix S3:** Measurement of PUFAs in maternal blood plasma phospholipids

Maternal blood samples were collected into heparinized tubes and plasma was extracted and stored at -80^o^C until analysis. Plasma lipids were extracted with chloroform–methanol (2:1, v/v). Phosphatidylcholine (PC), which contributes about 75% of plasma phospholipid, was isolated by solid phase extraction. Then, fatty acid methyl esters (FAMEs) were generated from PC after reaction with methanol containing 2% (v/v) sulphuric acid. FAMEs were extracted into hexane and separated by gas chromatography. FAMEs were identified by comparison with retention times of standards run previously, and they were quantified using ChemStation software (Agilent Technologies). Data were expressed as percentage contribution to the total plasma PC fatty acid pool. For all fatty acids within plasma PC, within-assay coefficient variation was <3 % and between-assay coefficient variation was <6 %.

**Appendix S4:** Measurement of cord blood cytokines

Cord blood eotaxin, interleukin-1 receptor antagonists (IL-1RA), interferon gamma induced protein 10 (IP-10), monocyte chemoattractant-1 (MCP-1), monokine induced by gamma (MIG), macrophage inflammatory protein-1 alpha (MIP-1alpha), macrophage inflammatory protein-1 beta (MIP-1beta), vascular endothelial growth factor A (VEGF-A), interleukin-12 subunit protein 40 (IL12-p40), plasminogen activator inhibitor-1 (PAI-1) and C-reactive protein (CRP) were measured using modified Luminex assay via DropArray (DA) multiplex assay (Curiox). Cord blood interleukin-10 (IL-10), interleukin-6 (IL-6) and tumor necrosis factor alpha (TNF-α) were measured using Human Cytokine 3-plex Quanterix SIMOA assay. All data were normalized and checked for plate effects using a median centering method.

**Appendix S5:** Statistical methods

Descriptive statistics for continuous variables are presented as mean (SD) when normality and homogeneity assumptions were satisfied, otherwise as median (IQR), and n (%) for categorical variables. Associations between natural log-transformed maternal PUFA ratios and total PUFA concentrations and allergic outcomes were assessed using Poisson regression, adjusting for demographic and relevant covariates. Adjusted associations between significant maternal PUFA ratios in Poisson regression and cord blood cytokines were performed using linear regression. The adjusted mediation effect of natural log-transformed cord blood cytokines on the associations between significant maternal PUFA ratios in Poisson regression and allergic outcomes were explored by mediation analysis.

**Reference**

1. Yi FC, Chew FT, Jimenez S, Chua KY, Lee BW. Culture of Blomia tropicalis and IgE immunoblot characterization of its allergenicity. *Asian Pac J Allergy Immunol*. 1999;17(3):189-194.
